# Design and Application of a Field Sensing System for Ground Anchors in Slopes

**DOI:** 10.3390/s130303739

**Published:** 2013-03-18

**Authors:** Se Woon Choi, Jihoon Lee, Jong Moon Kim, Hyo Seon Park

**Affiliations:** 1 Department of Architectural Engineering, Yonsei University, Seoul 120-749, Korea; E-Mails: watercloud@yonsei.ac.kr (S.W.C.); blithebard@naver.com (J.L.); 2 Dasumtek Incorporated, Seoul 222-22, Korea; E-Mail: dstek@dstek.biz

**Keywords:** strain sensing, wireless sensor node, residual tensile force, ground anchor

## Abstract

In a ground anchor system, cables or tendons connected to a bearing plate are used for stabilization of slopes. Then, the stability of a slope is dependent on maintaining the tension levels in the cables. So far, no research on a strain-based field sensing system for ground anchors has been reported. Therefore, in this study, a practical monitoring system for long-term sensing of tension levels in tendons for anchor-reinforced slopes is proposed. The system for anchor-reinforced slopes is composed of: (1) load cells based on vibrating wire strain gauges (VWSGs), (2) wireless sensor nodes which receive and process the signals from load cells and then transmit the result to a master node through local area communication, (3) master nodes which transmit the data sent from sensor nodes to the server through mobile communication, and (4) a server located at the base station. The system was applied to field sensing of ground anchors in the 62 m-long and 26 m-high slope at the side of the highway. Based on the long-term monitoring, the safety of the anchor-reinforced slope can be secured by the timely applications of re-tensioning processes in tendons.

## 1. Introduction

As the number of massive artificial slopes used for roads or residential area has increased, many cases of slope failures due to localized heavy rains, storms, and earthquakes continue to cause significant disasters [1,2]. To secure the structural safety of artificial slopes and to reduce risks of casualties or property damage, artificial slopes have been reinforced or supported by various methods (e.g., retaining walls, leaning walls, bioengineering slope protection, ground anchors, *etc.*). However, due to unexpected changes in local climate and imperfections in stability analysis or design of slopes, the risk of slope failures still remains. Consequently, besides the manual examination of the safety of slope at the site, various monitoring approaches have come to the forefront as alternatives for the assessment of the safety of various kinds of slopes including mountain slopes, mines, and cut slopes [3]. In the monitoring approaches, a variety of measuring instruments including extensometers, cameras, laser scanners, and GPS are used for monitoring of movements in slopes [4–7].

Among the various slope protection methods, a ground anchor system is one of the most frequently used methods for preventing the failure of a slope [8]. In a ground anchor system, cables or tendons connected to a bearing plate are used for stabilization of slopes. Therefore, for the case of steel ground anchors, the stability of a slope is dependent on maintaining the tension levels in the cables. The general process of inspections to maintain the intended performance of anchor-reinforced slope is majorly composed of several stages including simple inspections (e.g., daily inspections, periodical inspections) and precise inspections (e.g., lift-off test, ultrasonic testing). Especially, in order to check the state of ground anchor system, a process for evaluating tension levels (or residual forces) is commonly included in regular inspections according to the manuals [9–11].

However, when considering the situations of not only anchor-reinforced slopes but also typical slopes, a conventional monitoring system that is based on cable network is severely constrained. The most severe challenge is how the power is supplied and the monitoring system is maintained in an efficient way. The majority of slopes are located in places where access is inconvenient and electrical power is unavailable without additional electrical work. Furthermore, the sensing or monitoring system including lengthy cables and various devices is exposed to the risks of unexpected damages from lightning, rock-falls, and wildlife.

For these reasons, monitoring techniques utilizing wireless sensor networks (WSNs) have been proposed as a solution for various types of slopes. Jung *et al.* measured translation, rotation, and settlement of slope by two clinometers and one inclinometer [12]. This system is based on a ubiquitous sensor network utilizing local area wireless communication and mobile communication. He *et al.* developed a remote monitoring system for mining areas in order to prevent landslides through the measured sliding forces [13]. Song *et al.* developed a surface displacement monitoring system to observe changes in surface displacements and internal soil pressure [14]. Most research has dealt with monitoring of unreinforced slopes without tendons. So far, no research on field sensing or monitoring system for ground anchors has been reported. For the anchor-reinforced slope, a strain-based monitoring system is required to measure the residual force level in the anchors.

Therefore, in this study, a practical monitoring system for long-term monitoring of tension levels in tendons for anchor-reinforced slopes is proposed. In the monitoring system, the maximum tensile force in the tendon is measured by a wireless sensor node with vibrating wire load cells. The sensor nodes receive and process the measured signals before wirelessly transmitting them to higher level node (master node). Transmitted data is conveyed from the master node to a monitoring server in a remote office through code division multiple access (CDMA). The system was applied to field sensing of ground anchors in the 26 m high and 62 m long slope at the side of a highway. To test the performance of the proposed system, detailed measurement history during the total period of monitoring for 936 days (14 September 2009–6 April 2012) is provided and discussed.

## 2. Ground Anchor System

A ground anchor system in slopes is used to stabilize a slope and thus to prevent a slope failure. The purpose of the anchor system can be achieved by transferring the residual forces of anchors to the compression forces on ground. Since a pre-stressing technique was utilized on the Cheurfas Dam in Algeria in 1935, various forms of ground anchors have been developed and utilized in numerous structures, including bridges, buoyancy structures below ground water level, and tunnels, in addition to the slopes [8,15]. Nevertheless, most ground anchors have an identical basic mechanism of delivering residual force of a tendon to the ground.

**Figure 1.**
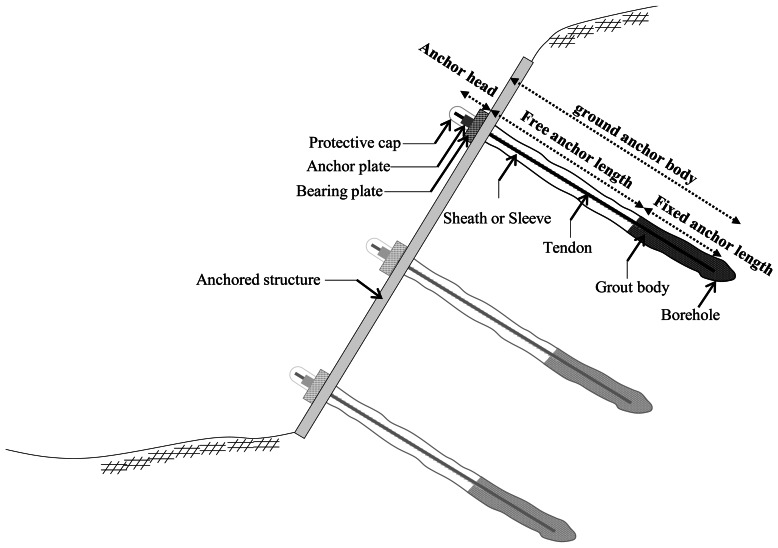
Components of ground anchor system.

All anchor systems have some of key common elements, as shown in Figure 1. According to EN 1537, a ground anchor is composed of three parts [9]: (1) ground anchor body (2) anchor head, and (3) relevant accessories. Ground anchor body is again divided into two parts: free anchor length and fixed anchor length. The part of free anchor length where strand or rod is covered by sheath delivers the residual force from anchor head to the part of fixed anchor length where tendon is grouted. A part of fixed anchor length again delivers residual force to ground by friction and compression. Depending on the types, ground anchor systems requires its own relevant accessories (e.g., wedge, nut and saddle of anchor head) to facilitate the operation of the mechanism.

The ground anchor is designed to avoid the possible failure mechanisms by considering: (1) overall stability of the anchor-reinforced slope, (2) inner stability of the anchor, and (3) stability of the bearing block. The overall stability of anchor-reinforced slope is assessed by structural analysis on the reinforcement effect of anchor on the predicted failure section. Various factors, including introduced residual force, decrease of residual force at installation, creep of the ground, and relaxation of tendon are considered in the analysis.

Securing the inner stability of the anchor is mandatory to prevent the occurrence of failure between grout body and ground, failure between grout and tendon, and tendon fracture. Also, the bearing block which serves the role of distributing the residual force of the anchor on the surface of slopes should not be destroyed by shear force or moment. During the service period, current states of reinforcement can be obtained through a monitoring system and compared with the intended design or expectation. The residual force of anchor on the reinforced slope is directly connected to various failure scenarios of system. Hence it becomes the main target of slope monitoring.

Monitoring of an anchor-reinforced slope aims to detect the overall or local deformation of slopes, to give the warning for the potential collapse of slope by evaluating the current states of reinforcement, and to provide informative data for maintenance and administration [16]. In actual cases of monitoring, a load cell is mainly utilized considering its great convenience in the installment and management. The load cell is installed on the anchor head and it measures the residual force of the ground anchor. If load cells are deployed in a real-time monitoring system, the change in residual forces is immediately measured when the slope is deformed.

After an initial residual force is applied, the residual forces of anchors tend to decrease according to the passage of time due to the creep of the ground or relaxation of steel, and afterward those forces are stabilized. However, various unexpected factors also have an influence on the fluctuation of residual force. The slope is deformed in accordance with both of problems in the ground anchor components (e.g., sub-standard quality of anti-corrosive materials, bad quality of anchors) and changes in the slope (e.g., freezing and thawing, expansion of ground) as fundamental factors [16]. In the worst case, the deformation intensifies a slope failure via damages of the anchor system. Since these damages of anchor system surely involve residual force changes, the monitoring system for detecting the residual force change can be used to draw consequent inspections. If the change of residual force is abnormal, an administrator launches subsequent reactions to identify an exact cause and seek well-timed maintenance. Also, an emergency warning can be given in case of a rapid slope failure predicted through the monitoring.

## 3. Monitoring System for Anchors in Slopes

**Figure 2.**
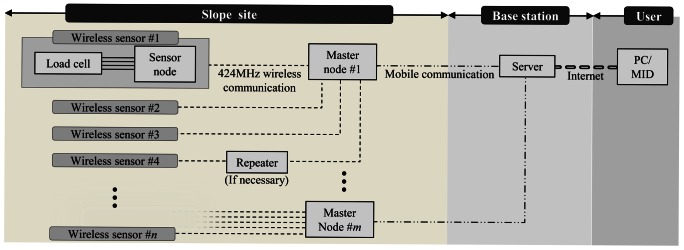
Field sensing system for anchor-reinforced slopes.

**Figure 3.**
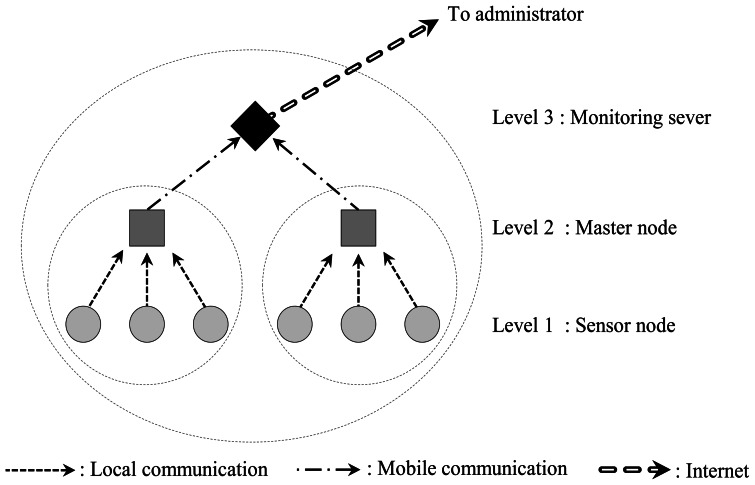
System topology for the monitoring of tendons in anchor slopes.

A practical monitoring system for anchor-reinforced slopes shown in Figure 2, and proposed in this paper, is composed of: (1) load cells which are based on VWSGs, (2) wireless sensing units (sensor nodes) which receive and process the signals from load cells and then transmit the result to a higher node (master node) through local area communication, (3) master nodes which transmit the data sent from sensor nodes to the server through mobile communication, and (4) a server located at a base station. As shown in Figure 3, single direction hierarchical sensor network architecture is adopted in the sensor network. For the end users, the monitoring software was designed to allow the access to tablet PC or smart phone as well as personal computers at wherever the internet service is available.

### 3.1. Load Cell and Sensor Node

**Figure 4.**
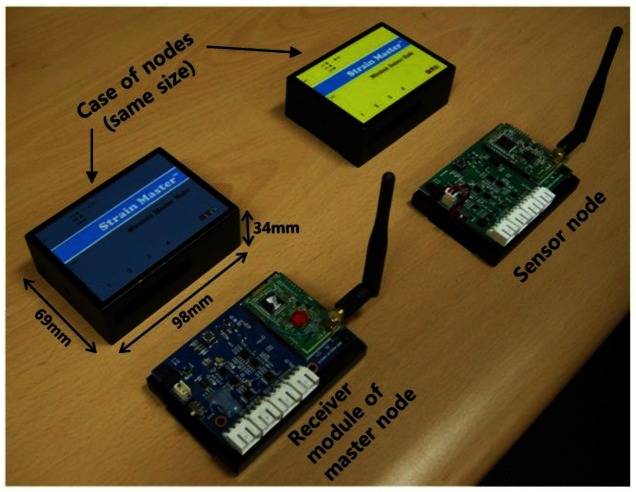
Sensor node and receiver module of the master node.

For measurement of the residual tensile forces of tendons in a ground anchor system, a VWSG-type load cell is developed and used in this paper, since VWSGs are not subject to electromagnetic interference (EMI) and have excellent endurance properties [17,18]. To reduce the influence of eccentricity in the tensile load acting on the load cell, multiple VWSGs need to be connected to the sensor node. Thus, as shown in Figure 4, the wireless sensor node for the load cell with 4-channel VWSGs consists of sensor process module, processor, memory, communication module, and power supply unit.

The sensor node inevitably requires batteries for the condition where a wired power supply is impossible. Thus, the low power consumption technology (LPCT) was introduced to resolve the power supply issue in management of nodes. The LPCT is realized by controlling active and inactive periods of a node, and this technique was named ‘active-sleep mode’. An active-sleep mode controls the nodes through two different modes: (1) active mode that makes a sensor node perform every function and (2) sleep mode that makes a sensor node inactive, which means that transmitting and receiving of the data is disabled, to minimize power consumption. By the active-sleep mode, a period of data acquisition can be manipulated and it has a direct influence in battery usage. In addition, transceiver and peripheral circuit are inserted into single chip of CC1020 transceiver of Chipcon, which is used in communication with 424 MHz UHF within the ISM bandwidth, to operate with low power consumption [19]. In this study, the sensor node is designed to consume the electric power of 50 mA in active mode and 200 μA in sleep mode. In addition, the sensor nodes are also operated by external DC power. Since most slopes are located in the optimal environment for harvesting the solar energy, the power supply issue can be resolved by utilizing solar battery.

### 3.2. Master Node, Repeater Node, and Monitoring Server

A master node serves the role of transmitting the data to the monitoring server located at a long distance by mobile communication after receiving the measured values from sensor nodes. If the communication between sensor nodes and a master node is not smooth due to obstacles on a slope, a repeater node is able to extend the distance of communication. Multiple wireless communications are possible between a master node and a number of sensor nodes.

A master node and repeater nodes also have a power issues. Thus, LPCT with active-sleep mode, which is already applied in sensor nodes, was also adopted in the master node and repeater nodes. If sensor nodes are in active mode, but master nodes or repeater nodes are in sleep mode, data cannot be transmitted to a monitoring server. Thus, the setting of active-sleep mode among sensor-repeater-master node should be in identical manner for successful transmission. Master nodes and repeater nodes can also get the electrical power from external DC power supply, a case similar to sensor nodes. The issue of power is efficiently resolved by utilizing solar battery and active-sleep mode.

The data between master node and the monitoring server located in base station is transmitted by a mobile communication technology. The base station positioned on-site decreases the applicability of monitoring system in a great manner due to burdensomeness in maintenance caused by difficult access, unexpected damages, and lastly the most severe issue of power supply. Hence, in this study, mobile communication technology is utilized so that the base station can be located at any place away from the site. The master node consists of two parts; a receiver module and CDMA transmitter. The receiver module of the master node shown in Figure 4 is directly connected with a commercially available CDMA transmitter through a short cable. After the receiver module receives and delivers the measurement data from the sensor nodes to the cable, the data is transmitted at the CDMA transmitter by means of the mobile communication, as shown in Figure 2. Thus a monitoring server can be located in a company providing monitoring service or in an office where slope administration is carried out.

### 3.3. Sensor Network System

The sensor network system for field monitoring of the ground anchors in Figure 2 is based on WCWNS (Web-Controlled Wireless Network Sensors) proposed by Mitchell *et al.* [20]. In the case of slopes with curved surfaces, even though most of the slopes are close to planes, an assumption that the surface of the curved slope is considered as a plane gives a great advantage in composing WSN.

**Figure 5.**
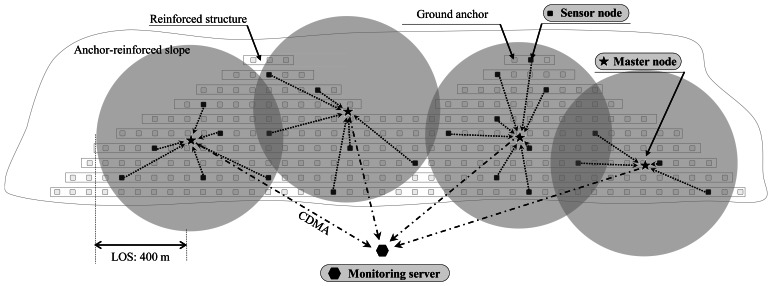
Sensor network configuration based on sensor nodes and master nodes deployed at field.

To make it possible, in this paper, wireless sensor nodes use the 424 MHz Ultra-High Frequency (UHF) band with good diffraction. It has a communication distance denoted by a line-of-sight (LOS) distance of about 400 m and a non-LOS distance of about 70 m with signal strength of 10 dBm. Therefore, as can be seen in Figure 5, a few master nodes are expected to sufficiently cover most of the slopes.

However, since the master node responsible for many sensor nodes acts as critical point during the communication process, circle representing the communication range of each master node in Figure 5 may be necessary to overlap as much as possible. In case of the malfunction of a master node, communications between the master node and sensor nodes can be replaced to adjacent master node in rapid manner. This process can be automated with peer-to-peer network architecture, but it would worsen the power consumption issue at field for anchor-reinforced slopes. Therefore, in this study, single directional communication from sensor nodes to the server in Figure 3 is adopted whereas replacing process of master nodes may be possible in the maintenance level.

### 3.4. Monitoring Software

**Figure 6.**
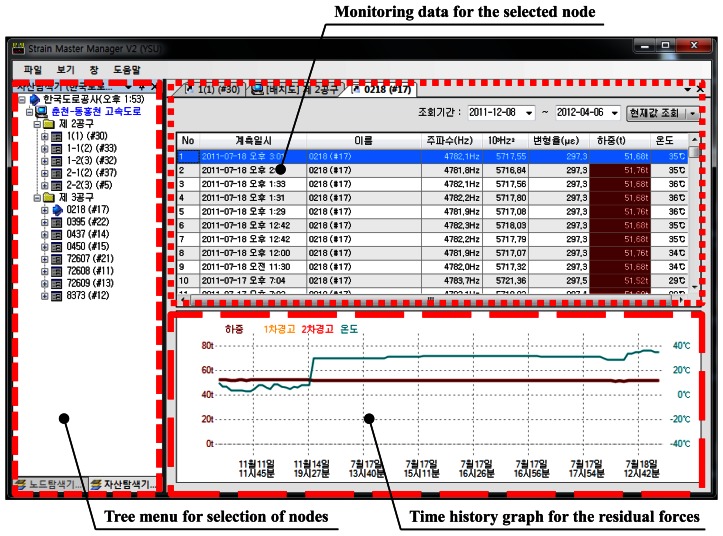
Screen capture showing measured data and the time history graph for residual forces.

Administrators will ultimately confirm the structural state of ground anchors via the monitoring software. The software for monitoring of ground anchors runs with World Wide Web and therefore anchors in slopes can be monitored remotely from any area where internet access is available. Figure 6 is an image of the software for personal computers currently in service. Its compositions are (1) sensor node explorer to select load cell, (2) monitoring data for the selected node, and (3) time history graph for the residual forces in the selected node. In addition, another version was also developed to allow the monitoring to take place in a tablet PC or a smartphone. Furthermore, when the slope reached dangerous stage, an alert system of sending cell phone messages and e-mails to related personnel was equipped.

## 4. Application to Field Sensing of Anchors in a Slope

**Figure 7.**
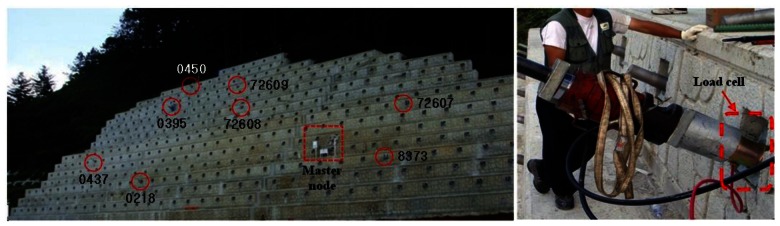
The target slope for field sensing of residual forces in ground anchors. (**a**) Perspective view of the slope. (**b**) Installation of a load cell for sensing of residual forces.

**Figure 8.**
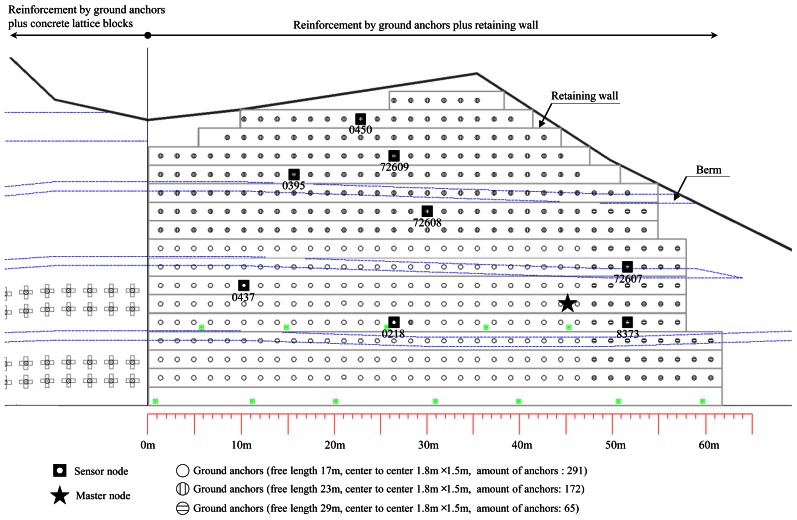
Master and sensor nodes for the field sensing of ground anchors at field.

The system was applied to field sensing of ground anchors in the slope at the side of a highway in Gangwon-do, Korea. The target slope is reinforced by ground anchors plus a concrete retaining wall, as shown in Figures 7(a) and 8. The length and vertical height of the slope in Figure 8 are approximately 62 m and 26 m, respectively. Three types of ground anchors were adopted for the slope reinforcement and total of 528 ground anchors were installed. Among them, eight ground anchors with different identification numbers indicated in Figure 7(a) were selected for field monitoring. The VWSG-based load cell installed on a ground anchor head is shown in Figure 7(b). As mentioned before, the residual tensile force of a tendon was calculated as the average value measured from 3 VWSGs.

**Figure 9.**
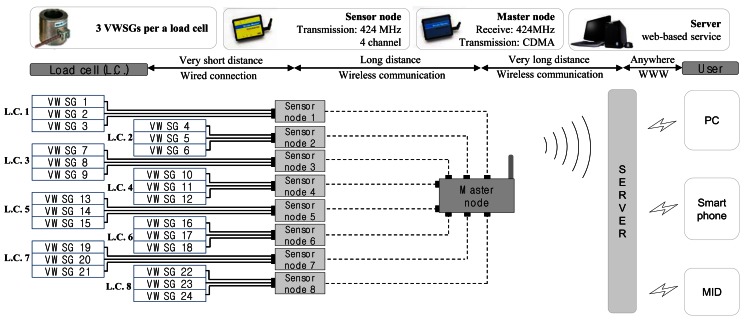
Sensor network system for the field monitoring of tensile forces in ground anchors.

The system for field sensing of residual tensile forces in ground anchors in the slope is shown in Figure 9. Since the length of the slope is shorter than the possible radius of local area communication (LOS = 400 m, non-LOS = 70 m) and there are no obstacles present within the communication radius, the usage of single master node in Figure 8 is sufficient for the field sensing. Then, as shown in Figures 8 and 9, the total of eight sensor nodes deployed at the slope transmit the data to the master node through local area wireless communication. Ultimately, the master node transmits the data to the monitoring server distanced far away from the site through CDMA communication and allows user to observe it via the web-based application. Sleep-active modes of sensor nodes and the master node are set to acquire a minimum of three data sets per day for the objective of low power consumption management.

**Figure 10.**
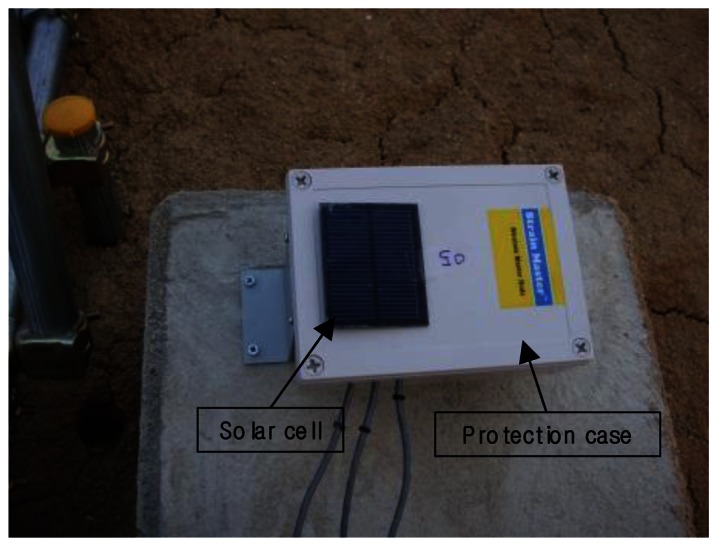
Solar cell and protection case for the sensor nodes at the field.

For supply of electric power to the nodes, a 3.7 V-2,700 mAh Li-Po battery was used in the site application. One battery enables a node to operate for approximately 270 days without charge since the sensor network system deployed in the site was designed to consume less than 10 mAh of electric power in one node daily. When considering that the slope is located in the area where solar energy can be supplied in stable manner, solar cells can effectively resolve the power issue which is one of the most severe problems in long-term operation in the field. As shown in Figure 10, maximum 5 V, maximum current of 1,000 mA, and maximum output of 0.5 W solar cells with the size of 60 mm × 75 mm were attached to protection cases of all sensor nodes. For the same reason, maximum 6 V, maximum current of 440 mA, and maximum output of 2.5 W solar cells with size of 120 mm × 180 mm was attached to the protection case of the master node.

**Figure 11.**
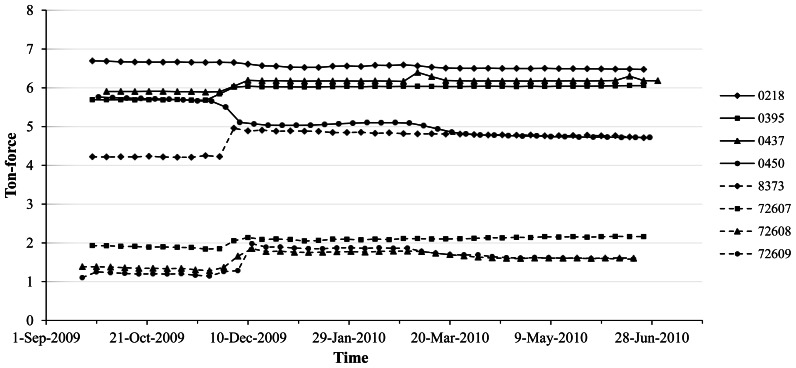
Time history graphs for weekly average residual forces measured from the system.

**Figure 12.**
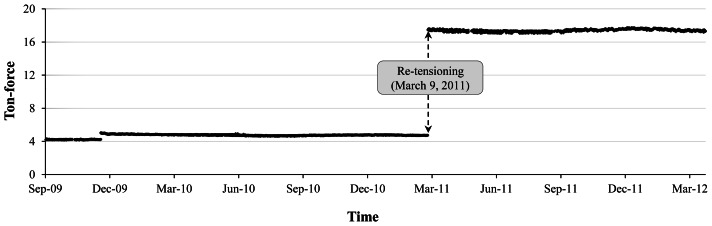
Time history graph before and after the re-tensioning work (anchor 8373).

**Figure 13.**
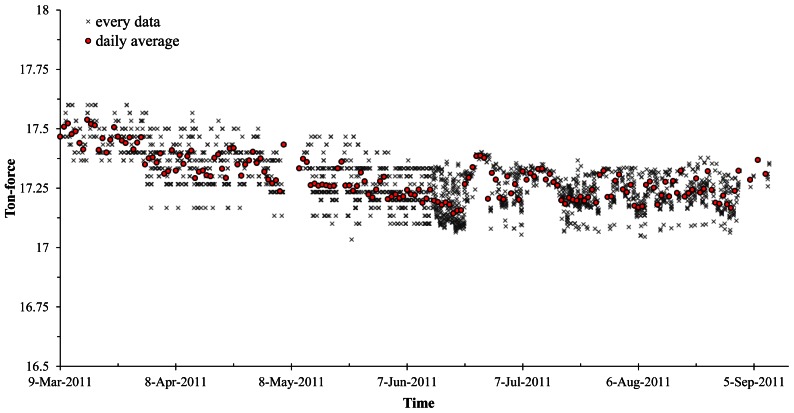
Monitored residual forces after the re-tensioning.

The load cells were installed on four separate dates of 9, 14, 17, and 21 in September 2009 and the monitoring started immediately after the load cells were installed on each anchor. Figure 11 shows graphs indicating weekly average values of residual force in tendons after installing the load cells for 40 weeks. The graphs showed gradual decrements in measured values for residual forces according to the passage of time. Based on the measured data, as clearly can be seen in Figure 12, the design level of the tensile residual force in the ground anchor with the identification number 8373 has been recovered by the application of re-tensioning processes at 9 March 2011. It is notable that the level of residual tensile forces in the tendon tends to decrease after the re-tensioning (Figure 13).

Although sleep-active modes were set to acquire minimum of three data sets per day by identical setting in all nodes, the active mode is occasionally converted to sleep mode without acquiring data as a result of jamming or malfunction. During the total period of monitoring for 936 days (14 September 2009–6 April 2012), the ground anchor with the identification number of 8373 showed several dates when the minimum three data sets were not acquired. For future study, this issue may be resolved by bidirectional communication between sensor nodes and the master node. Up to now, it was not applied in this system since the focus was on minimizing the power consumption.

**Figure 14.**
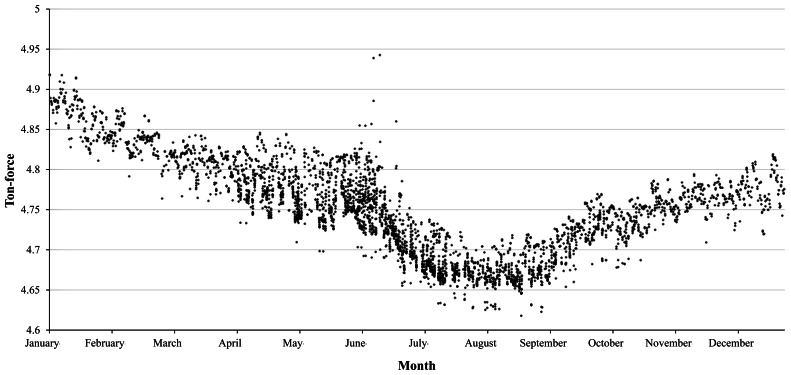
The change in residual forces of anchor 8373 for the year 2010.

To show the detailed measurements by proposed monitoring technology, the change in the residual tensile forces for the ground anchor with the identification number 8373 for a year of 2010 is presented in Figure 14. In the figure, the variations in the residual tensile forces coincide well with the variations in average monthly temperature for the site where the average monthly temperature reaches its maximum in summer (June to August). Also, for the year, the residual tensile force has been decreased by 2.23%. To secure the safety of the anchor-reinforced slope, a continuous and long-term monitoring of the tensile force in a tendon is necessary.

## 5. Conclusions

In a ground anchor system, one of the most frequently used methods for preventing the failure of a slope, cables or tendons connected to a bearing plate are used for slope stabilization. Then, the stability of a slope is dependent on maintaining the tension levels in the cables. Therefore, in this study, a practical sensing system for long-term monitoring of tension levels in tendons for anchor-reinforced slopes is proposed. The system was applied to field sensing of ground anchors in a 26 m high and 62 m long slope at the side of a highway. The history curves of the monitored data during the total period of monitoring for 936 days (14 September 2009–6 April 2012) showed significant decreases in the residual forces of anchors with the passage of time. Based on the long-term monitoring, the safety of the anchor-reinforced slope can be secured by the timely application of tendon re-tensioning processes.
